# This Time of Dying

**DOI:** 10.3201/eid1402.071511

**Published:** 2008-02

**Authors:** Polyxeni Potter

**Affiliations:** * Centers for Disease Control and Prevention, Atlanta, Georgia, USA

A must-read for those who think flu pandemic preparedness is much ado about nothing, this first novel by Reina James brings home the severity and plausibility of a flu disaster. And without data, statistics, or comment, it makes a compelling case for progress on the matter of influenza, to ease suffering, prevent spread, and possibly eliminate the scourge.

A series of journal entries beginning October 14 and ending November 7, 1918, this story of humans under stress unfolds in London against the savage backdrop of World War I and amidst the oft-recorded pestilence of Spanish flu. Without speculation, the author constructs population drama, one patient and one family at a time. The reader is drawn into it instantly and stays absorbed ’til the end.

Characters from all walks of life make up the cast of the unfolding tragedy. They chase about in their masks obsessing over work, social and self-imposed restrictions, unrelenting class barriers, and daily trivia until the schools close, the servants die or flee the ailing households, businesses fail, or until they succumb, filling the streets, mortuaries, graveyards, churches. We are allowed into their disinfected homes, to watch the banality, madness, or sheer horror of their lives. 

Some are stars. The heroic physician is back from the war with heightened awareness of the health emergency. “A plague is now among us which may well leave the earth to the animals,” he writes in a note intended to alert the health authorities. “You must stop the movement of troops, close our ports and warn others to follow suit.” He dies, “a blue man in the road,” before he can deliver the message. “His lips and ears were purple-blue, like a plum, and the skin on his face was mottled and pale but still tinged with blue, as if he’d been wiped with an inky rag.” 

The undertaker, who finds the warning note crumbled in the dead physician’s palm, is a man who plays the piano in between constructing coffins and practicing the family trade. He becomes increasingly unsettled by the note as the dead overwhelm his business, his city, his life. “The world’s body can hardly draw breath; it is sick and brought to its bed,” the physician had written. “Its wounds are open. The young spill out ... Death is crossing every sea.” He confirms that indeed the dying “were unusually young,” tries in vain to inform the authorities, and concludes with disbelief that “They had no plan.” The knowledge imparted by the note changes the undertaker’s life, which is already complicated by his universally disapproved affair with a woman “a little bit above his station.” 

His friend dismissed her, “She’s like a bloke.” Her friend, in a moment of weakness, thought her “a foolish ageing woman with a weak, offended face. Her lips merged unpleasantly into the skin surrounding them; her cheeks were dragged flat by failing muscle. There was too much eyelid and too little eyebrow. There was even a suggestion of fluff above the mouth, of the type that would inevitably grow into a moustache.” Their attraction, slowed by awkwardness and saved by spunk, adds an endearing almost hopeful quality to a tale rife with decomposing bodies. 

Another physician, a retired practitioner, is exhausted by the torrent of patients and his inability to improve their situation. Asked his opinion about the epidemic, he retorted angrily, “I haven’t got an *opinion *... I’m too busy.” Exasperated by people reporting “cyanosis,” he admitted that few things irritated him “more than patients using medical terminology.” In the absence of medical interventions, he suggested “fresh air in the bedroom, aspirin for headache, plenty of fluids and light food only, if tolerated.” His unloved wife and assistant by default props him up in bed to sign a few more death certificates before he collapses. 

Dedicated by the author to her maternal grandparents who died of it, this account of Spanish flu in London rings true for its hold on human behavior and of course for the flu, whose specter looms on our horizon. Along with the science, along with the vaccine, a dose of past history in human terms warns against underestimating a new pandemic. As James put it, “if every one of the newly bereaved were to hold a lantern in the sky, the man in the moon would think the world to be on fire.” 

